# The mediating role of psychological capital on the association between occupational stress and depressive symptoms among Chinese physicians: a cross-sectional study

**DOI:** 10.1186/1471-2458-12-219

**Published:** 2012-03-21

**Authors:** Li Liu, Ying Chang, Jialiang Fu, Jiana Wang, Lie Wang

**Affiliations:** 1 Department of Social Medicine, School of Public Health, China Medical University, 92 North 2nd Road, Heping District, Shenyang, 110001, People's Republic of China

## Abstract

**Background:**

Although occupational stress is an identified predictor of depressive symptoms, the mechanism behind the association is not well understood. The purpose of this study was to examine how psychological capital (PsyCap), a positive psychological state, mediates the association between occupational stress and depressive symptoms among Chinese physicians.

**Methods:**

A cross-sectional survey was conducted in Liaoning Province, China, during September–October 2010. Self-administered questionnaires including items on depressive symptoms assessed by the Center for Epidemiologic Studies Depression Scale, occupational stress assessed by the effort–reward imbalance scale and PsyCap estimated by a 24-item Psychological Capital Questionnaire, together with age, gender, marital status and education were distributed to 1300 physicians employed in large general hospitals. The final sample consisted of 998 participants. Asymptotic and resampling strategies were used to examine how PsyCap mediates the association between occupational stress and depressive symptoms.

**Results:**

Both the effort/reward ratio (ERR) and overcommitment were significantly associated with depressive symptoms among male and female physicians. There was a gender difference in the mediating role of PsyCap on the occupational stress–depressive symptoms association. For male physicians, PsyCap did not mediate the association between occupational stress and depressive symptoms. For female physicians, ERR and overcommitment were negatively associated with PsyCap, and PsyCap was negatively associated with depressive symptoms. As a result, PsyCap significantly mediated the associations of ERR and overcommitment with depressive symptoms. The proportion of PsyCap mediation was 19.07% for ERR, and 24.29% for overcommitment.

**Conclusions:**

PsyCap could be a positive resource for combating depressive symptoms in Chinese physicians. In addition to reducing occupational stress, PsyCap development should be included in depression prevention and treatment strategies, especially for female physicians.

## Background

Depression has become a major workplace mental health issue worldwide. Depression and depressive symptoms affect workers’ decision-making and cooperation [1,2], resulting in low productivity, absenteeism, job turnover, and economic costs [3,4]. In addition, depression and depressive symptoms can impair workers’ quality of life [5].

Physicians are usually exposed to a high level of occupational stress, and are at higher risk of suffering from depression than the general population and other low-risk occupational groups [6,7]. Our previous study found that the prevalence of depressive symptoms among Chinese physicians was 65.3% [8]. Frank and Dingle indicated that severe depression may even lead to more suicide attempts among physicians [9]. Depression and depressive symptoms are not only associated with physicians’ reduced work performance and professional responsibilities [10], but they also potentially threaten health care quality and patient safety. Fahrenkopf et al. reported that depressed residents made significantly more medical errors than their non-depressed peers [11]. Therefore, depression and depressive symptoms at work are a critical issue not only for the physicians themselves, but also for the health and safety of the patients they treat [8]. Thus, researchers around the globe have raised concerns about the prevention and treatment of physicians’ depression and depressive symptoms at work [8,12,13].

Occupational stress has been identified as a predictor of depression and depressive symptoms [12,14]. As a human service profession, physicians are highly exposed to various occupational stressors such as work overload, time pressures, role conflicts, effort–reward imbalance and unsatisfactory doctor–patient relationships [7,12,15]. Unfortunately, occupational stress is increasing in China because of the limited health workforce, patient-centered health care pattern and healthcare system reform. Therefore, Chinese physicians may be more vulnerable to depression and depressive symptoms compared with those in other occupational groups. Burnout, rational coping and workplace bullying significantly mediate the relations between occupational stress and depressive symptoms [16-18]. According to the results of previous studies, occupational stress not only exerts a direct effect but also has an indirect effect on depression and depressive symptoms through triggering specific psychological responses. Therefore, in-depth research on both direct and indirect effects of occupational stress on depression and depressive symptoms should be conducted to develop more effective strategies for depression prevention and treatment.

Positive psychological capital (PsyCap) is a higher-order core construct that fits within the positive organizational behavior approach, which is advocated for the study and application of positively oriented human resource strengths and psychological capacities [19]. PsyCap consists of the four state-like psychological resource capacities of self-efficacy, hope, optimism, and resilience, which can all be measured, developed, and effectively managed. Self-efficacy represents the positive belief about one’s abilities to succeed at challenging tasks. Hope is defined as a positive motivational state directing perseverance towards desired goals and pathways for success. Resilience is the positive psychological capacity to bounce back from (and beyond) failure and adversity to attain success. Optimism is a positive explanatory style regarding self-attributions for success [20]. PsyCap has significant positive effects on performance improvement [20-23], job embeddedness [22], satisfaction [20], organizational commitment [23], and well-being [24] in workplaces. PsyCap can also be used as a positive resource for combating employees’ stress symptoms and turnover [25]. In addition, PsyCap mediates the relation between supportive organizational climate and employee performance [26]. Luo and Hao reported that PsyCap mediated the relation between job burnout and turnover intention among Chinese nurses [27]. To our knowledge, the potential impact of occupational stress on PsyCap and the associations between PsyCap and depression and depressive symptoms have not been examined among health workers. In addition, whether or not PsyCap mediates the association between occupational stress and depression and depressive symptoms has not been confirmed. It is important to understand the effect of PsyCap on this association in order to effectively prevent and treat depression and depressive symptoms.

In light of the above concerns, the goals of the present study are to i) examine the association between occupational stress and PsyCap, ii) determine the association between PsyCap and depressive symptoms, and iii) investigate whether the association between occupational stress and depressive symptoms among Chinese physicians is mediated by PsyCap.

## Methods

### Study design and sample

A cross-sectional survey was conducted in Liaoning Province (population 43 million), China, during September–October 2010. According to the geographic distribution of Liaoning Province, one city in each region (eastern, western, southern, northern and central) was randomly selected. Two large general hospitals (> 500 beds) were randomly selected if the sampling city was a capital city; otherwise, one large general hospital was randomly selected from each city. In total, six large general hospitals were selected from five cities. We randomly sampled 50% of the physicians from each hospital. Self-administered questionnaires were directly distributed to 1300 physicians after obtaining written informed consent. Complete responses were obtained from 998 individuals (response rate: 76.77%), of whom 448 (44.89%) were males and 550 (55.11%) were females. The study was approved by the Committee on Human Experimentation of China Medical University, and the study procedures were in accordance with ethical standards.

### Measurement of depressive symptoms

Depressive symptoms were measured with the 20-item Chinese version of the Center for Epidemiologic Studies Depression Scale (CES-D) [28,29]. Each item contained four options that describe how often the respondents had each feeling in the past week, ranging from 0 ‘rarely or none of the time (less than 1 day)’ to 3 ‘most or all of the time (5 to 7 days)’. The summed score ranged from 0 to 60, which increased with the severity of depressive symptoms. The CES-D has been extensively validated in Chinese occupational groups [8,30]. For example, an acceptable Cronbach’s alpha of 0.88 was reported among health workers [30]. Cronbach’s alpha for the CES-D in the present study was 0.91 for both male and female physicians.

### Measurement of occupational stress

The effort–reward imbalance (ERI) model is built upon social reciprocity. Failed social reciprocity (high effort and low reward) at work elicits occupational stress [31]. With its focus on individual vital interests at work, the ERI model may be particularly suitable for studying the adverse health effects of occupational stress as currently witnessed in China. Specifically, the ERI model can explain how these adverse effects relate to work rewards that in turn directly affect the quality of life of Chinese people in the economic take-off stage. Chinese physicians experience failed social reciprocity and suffer from its health consequences [32,33].

The Chinese version of the ERI scale was translated and provided by Yang and Li [34]. The ERI scale consists of three subscales: effort (6 items), reward (11 items) and overcommitment (6 items). For the effort and reward subscales, the measurement procedure consists of two steps: initially, the participants express their attitude towards a work situation (‘agree’ or ‘disagree’); then, they are asked to evaluate to what extent (from ‘not distressed’ to ‘very distressed’) they usually feel distressed. For the overcommitment subscale, responses are scored from 1 to 4. From the ERI scale, occupational stress is expressed by two independent measures: effort/reward ratio (ERR) and overcommitment. The ERR is calculated using a predefined algorithm with a correction factor of 0.5454. An ERR beyond 1.0 indicates a high amount of effort spent that is not met with adequate reward. Those individuals who score high on overcommitment tend to spend an inadequate amount of effort that is not met by externally defined reward.

The Chinese ERI scale has been widely applied among Chinese occupational groups with good reliability and validity [32,33,35]. For Chinese physicians, Li et al. reported that the Cronbach’s alpha for the effort, reward and overcommitment subscales was 0.80, 0.83, and 0.74 for Chinese male physicians and 0.76, 0.80, and 0.78 for Chinese female physicians, respectively [33]. In this study, the Cronbach’s alpha for the effort, reward and overcommitment subscales was 0.91, 0.91, and 0.78 for male physicians and 0.90, 0.91, and 0.79 for female physicians, respectively.

### Measurement of psychological capital

The Chinese version of the 24-item Psychological Capital Questionnaire (PCQ) was used to measure PsyCap [20]. The PCQ consists of four subscales: self-efficacy (6 items), hope (6 items), resilience (6 items) and optimism (6 items). Each item has six responses with categories ranging from 1 ‘strongly disagree’ to 6 ‘strongly agree’. Because PsyCap is a higher-order core construct, the four key psychological resource capacities have a synergistic effect [26]. The average score for the total scale was calculated to get a composite PsyCap value in this study, with higher scores indicating more PsyCap.

Luthans et al. has demonstrated adequate reliability and construct validity of the PCQ across multiple samples [20]. The Chinese PCQ was initially applied among the Chinese population, including company employees [36,37], college students [38], and nurses [22,27]. Sun et al. reported a Cronbach’s alpha for self-efficacy, hope, resilience, optimism, and the total scale of 0.81, 0.82, 0.63, 0.50, and 0.88 for Chinese nurses, respectively [22]. After revision, Luo and Hao demonstrated that the Cronbach’s alpha for the total scale and subscales ranged from 0.71 to 0.93 [27]. In the present study, we conducted a confirmatory factor analysis that demonstrated adequate goodness-of-fit to the proposed second-order four-factor model (GFI = 0.900, TLI = 0.936, CFI = 0.945, and RMSEA = 0.063 for male physicians and GFI = 0.900, TLI = 0.921, CFI = 0.933, and RMSEA = 0.068 for female physicians). Cronbach’s alpha for self-efficacy, hope, resilience, and optimism in the current sample was 0.90, 0.89, 0.84, and 0.83 for male physicians and 0.88, 0.88, 0.85, and 0.85 for female physicians, respectively. For the total scale, the Cronbach’s alpha was 0.95 for male physicians and 0.94 for female physicians.

### Demographic characteristics

Demographic information regarding gender, age, martial status and education were obtained. Marital status was categorized as single or married. Education was categorized as junior college or lower, college, and graduate or higher.

### Statistical analysis

**Figure 1:**
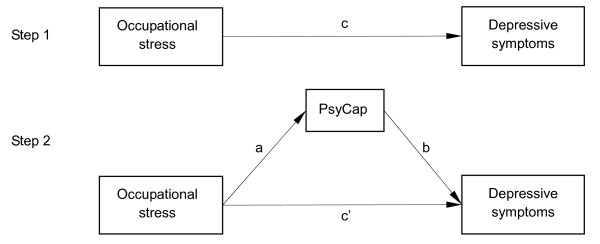
**Theoretical model of the mediating role of psychological capital on the association between occupational stress and depressive symptoms.** PsyCap: psychological capital. c: associations of ERR and overcommitment with depressive symptoms; a: associations of ERR and overcommitment with PsyCap; b: association between PsyCap and depressive symptoms after controlling for the predictor variables; c’: associations of ERR and overcommitment with depressive symptoms after adding PsyCap as a mediator.

Pearson’s chi-square (*χ*^2^) tests were used to compare differences in marital status and education among male and female physicians. Differences in continuous variables were examined by t-tests and one-way ANOVAs. Pearson’s correlation coefficients were used to examine correlations among continuous variables. We used the asymptotic and resampling strategies developed by Preacher and Hayes to examine PsyCap as a potential mediator on the association between occupational stress evaluated by the ERI scale and depressive symptoms [39]. In the regression equation, ERR and overcommitment were modeled as predictors, depressive symptoms as the outcome, PsyCap as the mediator (depicted in Figure 1), and age, martial status and education as covariates. The first step in the analysis was to determine the association between occupational stress and depressive symptoms (the c path) and the second was to estimate the mediating role (the a*b products) of PsyCap. When the c’ path coefficient in the second step was smaller than the c path coefficient in the first step, or was not significant, the possibility of mediation was speculated. The bootstrap estimate presented in our study was based on 5000 bootstrap samples. A bias-corrected and accelerated 95% confidence interval (BCa 95% CI) for each a*b product was investigated, and a BCa 95% CI excluding 0 indicated a significant mediation. All of the above analyses were conducted using SPSS for Windows (Ver. 13.0). Statistical significance was defined as p < 0.05. We analyzed the data for male and female physicians separately to allow for possible gender differences in the associations. All study variables were standardized before analysis to account for differences in scale scores.

## Results

### Participant characteristics

Marital status, education, age, and means and standard deviations of the study variables are presented separately for male and female physicians in Table 1. A minimum of college education was received by 90.32% of male and 90.36% of female physicians. There was a significant difference in marital status between male and female physicians (*χ*^2^ = 15.040, p < 0.01). Among the continuous variables, the only significant difference between the means for males and females was observed for age (t = 3.743, p < 0.01). Within each marital status and education group, there were no significant differences in scores for depressive symptoms, ERR, overcommitment, and PsyCap between male and female physicians. There were also no significant differences in the four variables across marital status and education groups.

**Table 1:** Participant characteristics, means and standard deviations (SDs) of variables

	**Males**						**Females**					
	**N (%)**	**Age**	**Depressive symptoms**	**ERR**^**†**^	**Over- commitment**	**PsyCap**	**N (%)**	**Age**	**Depressive symptoms**	**ERR**^**†**^	**Over- commitment**	**PsyCap**
		**Mean (SD)**	**Mean (SD)**	**Mean (SD)**	**Mean (SD)**	**Mean (SD)**		**Mean (SD)**	**Mean (SD)**	**Mean (SD)**	**Mean (SD)**	**Mean (SD)**
Total	448	35.92 (7.62)	19.54 (10.54)	0.88 (0.22)	16.43 (3.58)	4.18 (0.80)	550	33.98 (7.96)	19.46 (10.41)	0.85 (0.22)	16.39 (3.58)	4.15 (0.75)
Marital status												
Single	91 (20.31)	29.20 (5.33)	19.31 (11.80)	0.83 (0.20)	16.12 (3.35)	4.18 (0.84)	174 (31.64)	27.59 (5.34)	19.42 (9.92)	0.85 (0.22)	16.17 (3.24)	4.22 (0.69)
Married	357 (79.69)	37.63 (7.16)	19.60 (10.21)	0.90 (0.22)	16.54 (3.64)	4.18 (0.79)	376 (68.36)	36.94 (7.20)	19.48 (10.64)	0.85 (0.21)	16.48 (3.72)	4.13 (0.78)
Education												
Junior college or lower	43 (9.60)	39.45 (9.32)	20.27 (11.17)	0.90 (0.26)	15.82 (3.25)	4.08 (0.92)	53 (9.64)	38.02 (9.30)	21.16 (10.87)	0.87 (0.23)	15.82 (3.15)	4.05 (0.69)
College	274 (61.16)	36.19 (7.39)	20.11 (10.69)	0.87 (0.17)	16.53 (5.53)	4.13 (0.78)	311 (56.55)	34.45 (8.05)	19.53 (9.86)	0.85 (0.21)	16.26 (3.53)	4.15 (0.73)
Graduate or higher	131 (29.24)	34.15 (7.03)	18.10 (9.92)	0.90 (0.24)	16.43 (3.80)	4.30 (0.78)	186 (33.82)	32.04 (6.82)	18.85 (11.16)	0.85 (0.22)	16.75 (3.75)	4.21 (0.80)

ERR: effort/reward ratio; PsyCap: psychological capital

^†^ The mean of the ERR is logarithmic

### Correlations between study variables

Correlations between the study variables are presented in Table 2. For male physicians, age was positively correlated with overcommitment and PsyCap. Depressive symptoms were positively correlated with ERR and negatively correlated with PsyCap. Overcommitment was positively correlated with PsyCap. However, there were no significant correlations between depressive symptoms and overcommitment, or between ERR and PsyCap among males. For female physicians, age was positively correlated with PsyCap. All correlations between depressive symptoms, ERR, overcommitment, and PsyCap were significant among female physicians.

**Table 2:** Correlations between age, depressive symptoms, ERR, overcommitment and psychological capital

**Variables**	**Males**				**Females**
	**1**	**2**	**3**	**4**	**5**
1. Age	–	−0.027	−0.079	−0.060	0.089*
2. Depressive symptoms	−0.024	–	0.431**	0.174**	−0.375**
3. ERR	0.090	0.427**	–	0.512**	−0.321**
4. Overcommitment	0.134**	0.094	0.424**	–	−0.128**
5. PsyCap	0.101*	−0.325**	−0.039	0.106*	–

ERR: effort/reward ratio; PsyCap: psychological capital

*p < 0.05, **p < 0.01

### Psychological capital as a mediator of the association between occupational stress and depressive symptoms

Path coefficients, a*b products and BCa 95% CI for these products are presented in Table 3. First, the associations between occupational stress evaluated by the ERI scale and depressive symptoms (the c path) were determined. Positive associations of ERR and overcommitment with depressive symptoms were observed among both male and female physicians. The mediating role of PsyCap on the association between occupational stress and depressive symptoms was then estimated. For male physicians, ERR and overcommitment were not significantly associated with PsyCap (the a path). As a result, PsyCap did not mediate the association between occupational stress and depressive symptoms found in our study, even though PsyCap was significantly negatively associated with depressive symptoms (the b path) after controlling for the predictor variables. For female physicians, ERR (a = −0.301, p < 0.01) and overcommitment (a = −0.116, p < 0.01) were negatively associated with PsyCap. Consistent with the results of male physicians, PsyCap was significantly negatively associated with depressive symptoms after controlling for ERR and overcommitment. Thus, PsyCap significantly mediated the associations of ERR (a*b = 0.082, BCa 95% CI: 0.049, 0.126; p < 0.05; R^2^ = 0.251) and overcommitment (a*b = 0.043, BCa 95% CI: 0.005, 0.083; p < 0.05; R^2^ = 0.161) with depressive symptoms. The direct pathways between ERR, overcommitment and depressive symptoms (the c’ path) were still significant when PsyCap was included in the model as a mediator.

**Table 3:** Regression analysis results, with depressive symptoms as outcome and psychological capital as mediator

**Predictors**	**Path coefficients**				a*b (BCa 95% CI)	**R**^**2**^
	**c**	**a**	**b**	**c’**		
Males						
ERR	0.439**	−0.053	−0.290**	0.423**	0.015 (−0.028, 0.052)	0.284
Overcommitment	0.102*	0.093	−0.323**	0.132**	−0.030 (−0.071, 0.006)	0.125
Females						
ERR	0.430**	−0.301**	−0.272**	0.348**	0.082 (0.049, 0.126)	0.251
Overcommitment	0.177**	−0.116**	−0.368**	0.134**	0.043 (0.005, 0.083)	0.161

ERR: effort/reward ratio

c: associations of ERR and overcommitment with depressive symptoms; a: associations of ERR and overcommitment with PsyCap; b: association between PsyCap and depressive symptoms after controlling for the predictor variables; c’: associations of ERR and overcommitment with depressive symptoms after adding PsyCap as mediator; a*b: the product of a and b; BCa 95% CI: the bias-corrected and accelerated 95% confidence interval

Age, martial status, and education are covariates

*p < 0.05, **p < 0.01

To estimate the effect size of the mediating pathway, we calculated the proportion of the total effect of the independent variable on the dependent variable (c) that was mediated by PsyCap with the formula (a*b)/c. For female physicians, the proportion of PsyCap mediation was 19.07% for ERR, and 24.29% for overcommitment.

## Discussion

As a special occupational group, physicians experience more severe occupational stressors than most other professional groups, such as employees from the education service, machinery, equipment, and electronic components manufacturing, public agency and national defense [7,40]. The duty of physicians is to save lives. Thus, they are highly exposed to suffering, disease and even death every day. They face high demands and inevitably bear more responsibility and experience greater uncertainty than other professions. In this study, the total effects of extrinsic stress (ERR) and intrinsic stress (overcommitment) evaluated by the ERI scale on depressive symptoms were significant among both male and female physicians. These results are consistent with previous studies of health workers from different countries [30,41]. Therefore, occupational stress reduction should be included in interventions for depression prevention and treatment targeted at Chinese physicians. On one hand, hospital managers should balance the workload and work reward (such as work hours, fair work allocation, promotion, stability, respect, support, and income) of physicians. On the other hand, physicians should avoid overcommitment through developing effective strategies for managing time, taking on suitable commitments, focusing on efforts for coping with work demands, and learning effective methods for getting things done.

To develop effective interventions, the roles of positive predictors and mediators should be explored to clarify the mechanism behind the association between occupational stress and depressive symptoms. Practical strategies for developing employees’ PsyCap would help them better cope with various occupational stressors. In the present study, a negative association between PsyCap and depressive symptoms among both male and female physicians was confirmed. Adequate levels of PsyCap may alleviate depressive symptoms. It was implied that PsyCap could mediate the association between occupational stress and depressive symptoms.

As a capacity that can be modified and developed, an individual’s level of PsyCap might be affected by various workplace factors. PsyCap is significantly negatively associated with the symptoms of stress and job stress (from workload, career development, interpersonal relationships and work-family balance) [25,37]. In the present study, we found a significant negative association between occupational stress and PsyCap among female, but not male physicians. These results indicate a gender difference in the association between occupational stress evaluated by the ERI scale and PsyCap among Chinese physicians. Reasons for this difference should be considered. Females may be more sensitive to perceptions of occupational status than males. Hence more serious consequences might occur among females compared with males experiencing the same level of occupational stress [42]. Moreover, female physicians confront more workplace adversity than males in terms of disdain, mistrust from the patients, and uncertain job prospects, as well as having the dual responsibilities of career and family. This leads females to pay more attention to their vested interests such as effort and reward, and thus affecting their PsyCap level at work. For male physicians, they generally have lower levels of caregiving burdens and family obligations than females. They can dedicate themselves whole-heartedly to their careers. Furthermore, they generally pay more attention to individual career development [43]. As a result, it is suggested that future research should focus on gender differences in the antecedents to workplace PsyCap to investigate gender-appropriate PsyCap development strategies and practices.

This is the first study to confirm the mediating role of PsyCap on the association between occupational stress and depressive symptoms among Chinese female physicians. High extrinsic (ERR) and intrinsic (overcommitment) stress might reduce PsyCap levels, resulting in depressive symptoms in female physicians. As mentioned previously, PsyCap can be developed through a variety of ways when its four constructs are combined into a higher-order construct [44,45]. Therefore, our results have practical significance for Chinese physicians, especially females. Based on the results of this study, those previously developed strategies for increasing PsyCap should be applied to Chinese physicians to improve their PsyCap level and relieve depressive symptoms.

In spite of these merits, two limitations of the present study are acknowledged. First, our study was cross-sectional, and thus we are unable to assess the causal relations among study variables. However, our study hypotheses were built on solid theoretical and research foundations. The results from our cross-sectional study need to be confirmed with a prospective cohort study. Second, the study population comprised physicians from large general hospitals only. A previous study in China found that physicians from large general hospitals experienced more severe depressive symptoms than those from smaller hospitals [46]. Our sample represented the majority of working physicians from large general hospitals in China. However, it is not clear whether the identified mediating role of PsyCap on the association between occupational stress and depressive symptoms could be extended to other occupational groups.

## Conclusions

Both extrinsic stress (ERR) and intrinsic stress (overcommitment) evaluated by the ERI scale were significantly associated with depressive symptoms among male and female physicians. For male physicians, ERR and overcommitment were not associated with PsyCap. Thus, for males, PsyCap did not mediate the association between occupational stress and depressive symptoms, even though PsyCap was negatively associated with depressive symptoms. For female physicians, ERR and overcommitment were negatively associated with PsyCap, and PsyCap was negatively associated with depressive symptoms. Thus, PsyCap partially mediated the association between occupational stress and depressive symptoms. In addition to reducing the level of occupational stress through balancing workloads, increasing work rewards, and avoiding overcommitment, PsyCap development should be included in prevention and treatment strategies for depression targeted at Chinese physicians, especially females.

## Competing interests

The authors declare that they have no competing interests.

## Authors’ contributions

LL designed the research, carried out data analysis and wrote the paper. LW provided guidance in study design, organized the investigation and is the corresponding author. YC and JLF provided help with the data collection, analysis, interpretation and write-up. JNW provided help with the data collection and interpretation. All authors read and approved the final manuscript.

## Pre-publication history

The pre-publication history for this paper can be accessed here:

http://www.biomedcentral.com/1471-2458/12/219/prepub
